# 774. Trivia Game for Learning Zoonotic Diseases in Pre-clinical Medical Curriculum

**DOI:** 10.1093/ofid/ofad500.835

**Published:** 2023-11-27

**Authors:** Celeste L Moreland, Catherine Henry, Adrienne Carey

**Affiliations:** University of Utah School of Medicine, Salt Lake CIty, Utah; University of Utah School of Medicine, Salt Lake CIty, Utah; University of Utah School of Medicine, Salt Lake CIty, Utah

## Abstract

**Background:**

Zoonotic diseases are high-yield material, frequently tested on USMLE Step 1. For learning infectious diseases, many medical students supplement or replace coursework with outside resources that are personalizable and utilize active learning^1,2^, but these resources can be prohibitively expensive, have questionable reliability, and align imperfectly with course content^2^. Spencer Fox Eccles SOM historically used a Team-Based Learning (TBL) format for teaching zoonotic diseases, but this content is primarily memorization-based, while TBL is well suited for conceptual material. To teach zoonotic diseases, we created a trivia game^3^ (“ZooTrivia”) to mimic the low-stakes, fast-paced, engagement of outside resources, while still capturing the cooperative learning environment of TBL.

**Methods:**

We used the Kirkpatrick model for student reaction and learning to assess efficacy of ZooTrivia. The session was opt-in and students chose their own teams to answer trivia questions. All answers were provided at the end. We used attendance data and surveys to assess student enjoyment of and perceived learning from ZooTrivia.

**Results:**

Twenty (20/125) students reported attending ZooTrivia. Three students, who do not usually attend class, chose to attend ZooTrivia, which suggests that trivia is an attractive format. 60% of attendees reported that they would like more games like this in their education. 30% responded that they would “maybe” want more games like this. For enjoyment, students on average reported preferring trivia over both TBL and outside resources (average 3.9 and 3.6, respectively on a scale of 1-5). However, for student learning, students on average reported TBL and outside resources as better learning formats (average 2.8 for both, on a scale of 1-5).

**Conclusion:**

Trivia is an alternative to TBL-based learning of zoonotic disease that students report enjoying more but learning slightly less than TBL. Analysis of student learning was limited by incomparable attendance of ZooTrivia and TBL; ZooTrivia was an optional session, while TBL was required attendance. This comparison is a direction for future study.

**Disclosures:**

**All Authors**: No reported disclosures

